# Epidemiology of type 1 and type 2 diabetes mellitus in Kazakhstan: data from unified National Electronic Health System 2014–2019

**DOI:** 10.1186/s12902-022-01200-6

**Published:** 2022-11-11

**Authors:** Dinara Galiyeva, Arnur Gusmanov, Yesbolat Sakko, Alpamys Issanov, Kuralay Atageldiyeva, Kainar Kadyrzhanuly, Aiymzhan Nurpeissova, Marzhan Rakhimzhanova, Aigul Durmanova, Antonio Sarria-Santamera, Abduzhappar Gaipov

**Affiliations:** 1 Department of Medicine, School of Medicine, Nazarbayev University, Kerey and Zhanibek street #5/1, Nur-Sultan, Kazakhstan 010000; 2 School of Population and Public Health, University of British Columbia, Vancouver, BC Canada; 3 Clinical Academic Department of Internal Medicine, University Medical Center, Nur-Sultan, Kazakhstan; 4 Department of Medical Information Analysis of Outpatient and Polyclinic Care, Republican Center of Electronic Healthcare, Nur-Sultan, Kazakhstan; 5 Clinical Academic Department of Pediatrics, University Medical Center, Nur-Sultan, Kazakhstan

**Keywords:** Diabetes, Survival, Mortality, Epidemiology, Comorbidities

## Abstract

**Background:**

We aimed to explore descriptive epidemiology of T1 and T2 Diabetes Mellitus (DM) and to investigate demographic factors and comorbidities associated with all-cause mortality by aggregating and utilizing large-scale administrative healthcare data from the Unified National Electronic Health System (UNEHS) of Kazakhstan for 2014–2019 years period.

**Methods:**

A total of 475,539 individuals were included in the analyses. The median years of follow-up for Type 1 DM patients accounted for 4.7 years and 4.5 years in Type 2 DM patients. We used Kaplan-Meier and log-rank test to calculate failure function and differences in survival by age, sex, ethnicity, and comorbidities with all-cause mortality for Type 1 and Type 2 DM. Cox proportional hazards regression analysis was used to obtain crude and adjusted hazard ratios.

**Results:**

Prevalence of Type 1 and Type 2 DM increased 1.7 times from 2014 to 2019. Mortality of Type 1 and Type 2 DM also increased 4 times and 6 times from 2014 to 2019, respectively. Male sex, older age and Kazakh ethnicity were associated with a higher risk of all-cause death compared to females, younger age and other nationalities than Kazakh in patients with Type 1 and Type 2 DM. Coronary artery disease, diabetic nephropathy, stroke, amputations and neoplasms were associated with a higher risk of all-cause death.

**Conclusion:**

The prevalence and mortality rate of Type 1 and Type 2 DM increased during the years 2014–2019 in Kazakhstan. Male sex, older age and Kazakh ethnicity were associated with a higher risk of all-cause death compared to females, younger age and other nationalities than Kazakh. Coronary artery disease, diabetic nephropathy, stroke, amputations and neoplasms were associated with a higher risk of all-cause death.

**Supplementary Information:**

The online version contains supplementary material available at 10.1186/s12902-022-01200-6.

## Introduction

Over the past few decades, the prevalence of diabetes in developed and developing countries has risen substantially, making diabetes a major public health issue worldwide. The global prevalence of diabetes in 2019 was estimated to be 9.3% (463 million people), rising to 10.2% (578 million) by 2030 and 10.9% (700 million) by 2045 [1]. The prevalence is higher in urban (10.8%) than rural (7.2%) areas, and in high-income (10.4%) than low-income countries (4.0%) [1].

Although, majority of the data about prevalence, incidence, and all-cause mortality rates in patients with diabetes obtained from high-income countries, there is no published data available for the epidemiology of type 1 and type 2 diabetes in low- and middle-income Central Asian countries, including Kazakhstan. Couple of small-scale observational studies done to explore diabetes epidemiology in Kazakhstan [2–5]. A cross-sectional study conducted in four geographically remote regions of Kazakhstan using the WHO STEP survey among 4753 participants showed the survey-weighted prevalence of impaired fasting glycemia (IFG) 1.9% and of T2DM 8.0% [4]. However, there is no study investigated DM epidemiology using national health registry data in Kazakhstan before.

To date, Kazakhstan has well developed electronic healthcare system among many other countries [6, 7]. This is so called Unified National Electronic Health System (UNEHS), which started in 2003 and established in 2014, made available medical claims from different electronic data sources such as inpatient electronic registries of hospitalized patients, outpatients electronic registries of dispensary patients and many others, which are implemented overall all medical organizations in the country. Aggregation and utilization of such nationwide electronic healthcare data could provide real knowledge on in-depth epidemiology of diabetes in overall Kazakhstan [6, 7].

Therefore, we aimed to explore descriptive epidemiology of T1DM and T2DM and to investigate demographic factors associated with all-cause mortality among T1DM and T2DM. Moreover, we aimed to determine comorbidities associated with all-cause mortality among T1DM and T2DM patients in Kazakhstan by aggregating and utilizing large-scale administrative healthcare data from the Unified National Electronic Health System (UNEHS) for 2014–2019 years period.

## Materials and methods

### Data setup and study population

This is a retrospective study, including all Type 1 and Type 2 DM patients identified through the UNEHS, who were registered with DM between 2014 and 2019. The UNEHS consists of inpatient and outpatient electronic registries, which collect individual patient data on socio-demographics and clinical data based on International Classification of Diseases 10 (ICD-10) codes (see Supplementary Material Table 1).

**Fig. 1:**
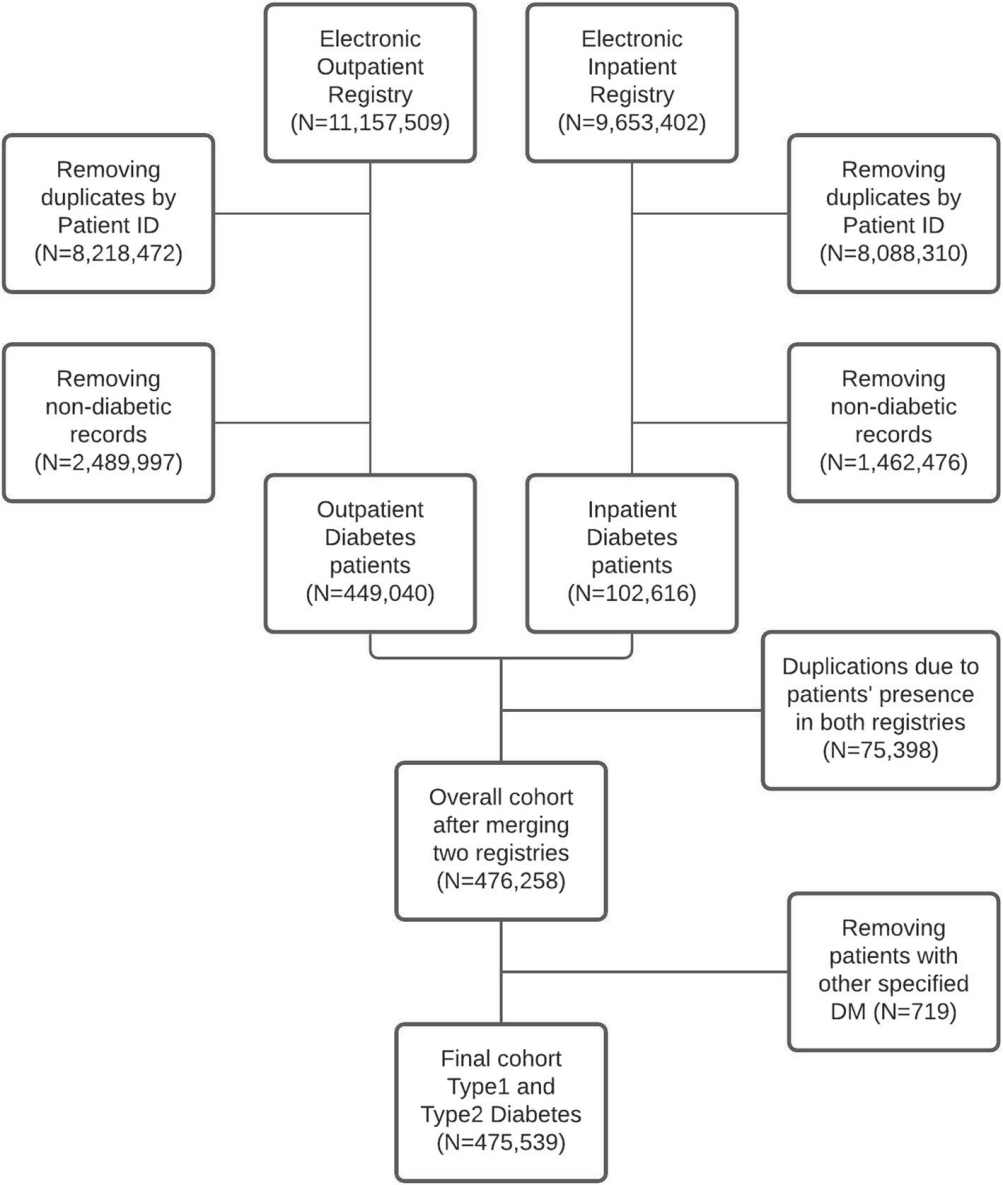
Flow chart of the cohort selection process from the Unified National Electronic Health System (UNEHS)

Initial data consisted of 20,810,911 patient records from inpatient electronic registry of hospitalized patients (*n* = 9,653,402) and outpatient electronic registry of dispensary patients (*n* = 11,157,509) which were identified using ICD-10 codes for DM (E08 – E13) [6]. After removing duplicates and non-diabetic patients, the final cohort consisted of 475,539 DM patients. For more details about the study sample selection process refer to Fig. 1. Flow chart of the cohort selection process from the Unified National Electronic Health System (UNEHS).

### Exposures and covariates

Individual patient data included demographical variables such as date of birth, sex, address, ethnicity and disease related information including ICD-10 codes for main diagnosis, comorbidities and complications, ICD-9 codes for procedures and surgical operations [8], admission/discharge dates and first date of diagnosed diabetes. Information about date of death (if any) were obtained through linkage with the Population Registry through unique population registry number (RPN number) [9].

Age was categorized to: 18 years old (y.o.) and below, 19–34 y.o., 35–50 y.o., 51–70 y.o. and above 71 y.o.. Ethnicity was grouped to Kazakhs, Russians, and others (included Uzbeks, Uyghurs, Ukrainians, Koreans, and other 37 ethnicities).

Comorbidities and diabetes related complications were obtained from existing primary and secondary ICD-10 codes available in the overall cohort. The following important variables included: coronary artery disease, hypertension, stroke, diabetic retinopathy, diabetic nephropathy, diabetic neuropathy, diabetic foot, amputations, and neoplasms. Definition of each above-mentioned variables are provided in Supplementary materials Table 1.

### Outcome assessment

Patients with ICD-10 codes E10 and E11 were defined as Type 1 and Type 2 DM, respectively. The prevalence, incidence and all-cause mortality of Type 1 and Type 2 DM patients were assessed for six consecutive years (2014–2019). For each year, a period prevalence was calculated by dividing all alive Type 1 and Type 2 DM patients at any point of the year by the average total general population size during the year. Similarly, incidence and mortality were calculated by dividing the number of new patients and deaths, respectively, by the average total general population size for each year. The number of population growth in Kazakhstan overall and its regions were obtained from the Statistics Committee [10].

All-cause mortality data were obtained from the National Population Registry [11]. The start of the follow-up period was the date of DM diagnosis, and patients were followed up until death or or end of the follow-up (December 31st, 2019), whichever came first.

### Statistical methods

Data are summarized as percentages for categorical variables and as median and interquartile range (IQR) for continuous variables. We used Kaplan-Meier and log-rank test to calculate failure function and statistically significant differences in survival by age, sex, ethnicity, and comorbidities with all-cause mortality for Type 1 and Type 2 DM. Cox proportional hazards regression analysis, after checking its assumptions, was used to obtain crude and adjusted hazard ratios in different models. In Model 1 adjustments were made for sex, age categories and ethnicity. In Model 2 additional adjustments were for sex, age categories, ethnicity, diabetic neuropathy, coronary artery disease, hypertension, diabetic nephropathy, retinopathy, stroke, diabetic foot, amputations, and neoplasms. The crude incidence and prevalence rates for Type 1 and Type 2 DM per 100,000 population were also evaluated. The crude mortality rates were evaluated per 100,000 population and per 1000 Person-Years.

All statistical analyses were performed using STATA 15 MP2 Version (STATA Corporation, College Station, TX). *P* values are two- sided and reported as statistically significant at < 0.05 for all analyses. Due to the retrospective nature of the study, the Institutional Review Ethics Committee of Nazarbayev University has waived the need for informed consent and approved this study (NU-IREC 490/18112021). The study was performed according to both international local ethics guidelines and regulation as well as declaration of Helsinki.

## Results

### Patient characteristics

Table 1 presents the demographic characteristics of the 475,539 registered Type 1 and Type 2 DM patients (31,763 and 443,776 patients, respectively). More females (64.2%) than males were observed among patients with Type 2 DM, while opposite was observed among Type 1 DM patients. Almost half of each cohort of diabetic patients were Kazakh. Type 2 DM patients were older than patients with Type 1 DM with median age at diagnosis being 26.9 (IQR 13.4–43.5) and 58.5 (IQR 51.3–65.8) ages, respectively. The following comorbidities and diabetes-related complications were more common in Type 1 DM patients compared to Type 2 DM: diabetic neuropathy (11.4% vs 5.3%), nephropathy (4.9% vs 1.5%), retinopathy (10.9% vs 3.6%), diabetic foot (9.5% vs 3.8%) and neoplasms (7.2% vs 4.9%), while hypertension (44.3% vs 21.7%) and coronary artery disease (18.9% vs 16.4%) were more common in Type 2 DM patients compared to Type 1 DM. Relative frequencies of those who died among Type 1 and Type 2 DM patients were comparable – 11.3% (3581 deaths) and 13.7% (60,570 deaths), respectively.

**Table 1:** Baseline characteristics of Type 1 and Type 2 DM patients registered between 2014 and 2019

Characteristics	Total ***N*** = 475,539	Type 1 diabetes mellitus patients ***N*** = 31,763	Type 2 diabetes mellitus patients ***N*** = 443,776
**Sex, Female**	300,268 (63.2%)	15,262 (48.1%)	285,006 (64.2%)
Median (IQR) age when diagnosed	57.8 (49.8–65.3)	26.9 (13.4–43.5)	58.5 (51.3–65.8)
**Age groups**			
< =18	12,688 (2.7%)	11,562 (36.4%)	1126 (0.3%)
19–34	17,866 (3.8%)	8646 (27.2%)	9220 (2.1%)
35–50	103,199 (21.7%)	5798 (18.3%)	97,401 (21.9%)
51–70	282,678 (59.4%)	4761 (15.0%)	277,917 (62.6%)
> =71	59,108 (12.4%)	996 (3.1%)	58,112 (13.1%)
**Ethnicity**			
Kazakh	227,611 (47.9%)	14,843 (46.7%)	212,768 (47.9%)
Russian	137,229 (28.9%)	8057 (25.4%)	129,172 (29.1%)
Other	107,434 (22.6%)	6899 (21.7%)	100,535 (22.7%)
Missing	3265 (0.7%)	1964 (6.2%)	1301 (0.3%)
**Outcome**			
Median duration of diabetes, years (IQR)	4.5 (2.1–7.9)	4.7 (2.2–8.2)	4.5 (2.1–7.8)
Duration of diabetes, N(%)			
< 5 years	260,267 (54.7%)	16,882 (53.2%)	243,385 (54.8%)
5–10 years	140,480 (29.5%)	8750 (27.6%)	131,730 (29.7%)
> 10 years	74,792 (15.7%)	6131 (19.3%)	68,661 (15.5%)
Dead	64,151 (13.5%)	3581 (11.3%)	60,570 (13.7%)
Median age at death, years (IQR)	70.6 (62.9–78.8)	61.1 (42.6–71.6)	71.0 (63.5–79.0)
**Comorbidities/complications**			
Diabetic neuropathy, yes	27,074 (5.7%)	3626 (11.4%)	23,448 (5.3%)
Coronary artery disease, yes	88,931 (18.7%)	5223 (16.4%)	83,708 (18.9%)
Hypertension, yes	203,592 (42.8%)	6890 (21.7%)	196,702 (44.3%)
Diabetic nephropathy, yes	8118 (1.7%)	1551 (4.9%)	6567 (1.5%)
Diabetic retinopathy, yes	19,269 (4.0%)	3472 (10.9%)	15,797 (3.6%)
Stroke, yes	11,846 (2.5%)	888 (2.8%)	10,958 (2.5%)
Diabetic foot, yes	19,974 (5.4%)	3020 (9.5%)	16,954 (3.8%)
Amputations, yes	4749 (1.0%)	1156 (3.6%)	3593 (0.8%)
Neoplasms, yes	27,142 (5.1%)	2274 (7.2%)	21,868 (4.9%)

### Incidence, prevalence and mortality rates

**Fig. 2:**
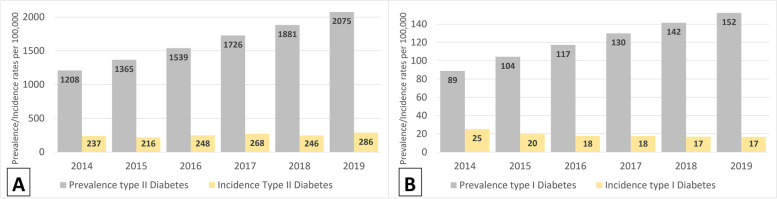
Prevalence and incidence per 100, 000 population/years ((**A**) - Type 2 DM patients, (**B**) - Type 1 DM patients)

**Fig. 3:**
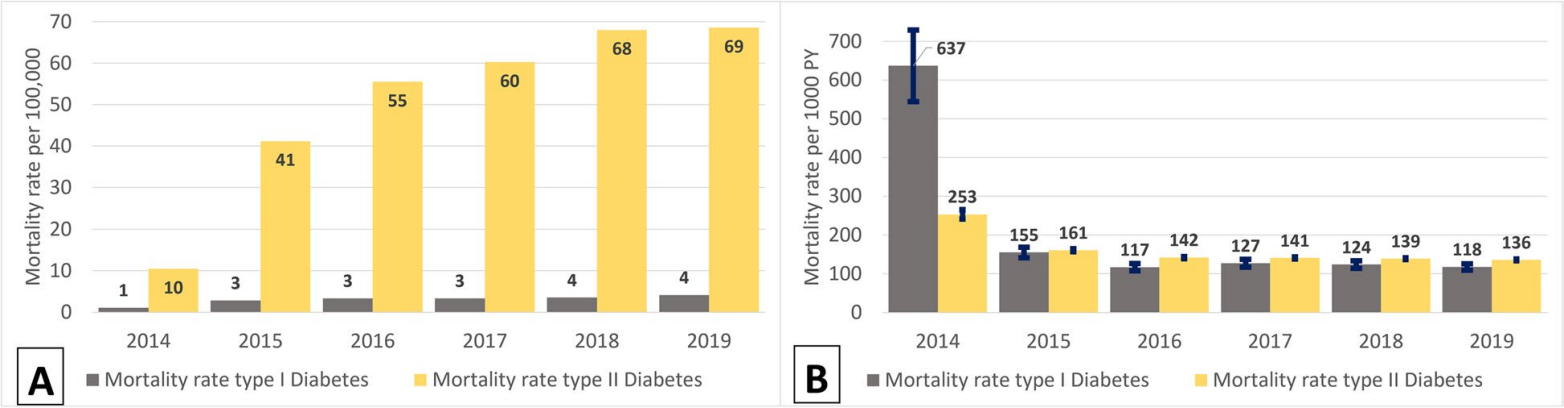
Mortality rate per 100, 000 population/years (**A**) and per 1000 person-years (PY) with 95% CI (**B**)

The prevalence of both Type 1 and Type 2 DM increased almost two times in 2019 compared to 2014, from 86 to 152 per 100,000 for Type 1 DM and from 1208 to 2075 per 100,000 for Type 2 DM (Fig. 2. Prevalence and incidence per 100, 000 population/years ((A) - Type 2 DM patients, (B) - Type 1 DM patients).. Incidence of Type 1 DM decreased 1.5 times from 2014 to 2019, while incidence of Type 2 DM was similar over the years (Fig. 2. Prevalence and incidence per 100, 000 population/years ((A) - Type 2 DM patients, (B) - Type 1 DM patients).). Despite increase in mortality of diabetic patients in a whole population during 5 years (Fig. 3. Mortality rate per 100, 000 population/years (A) and per 1000 person-years (PY) with 95% CI (B).a), mortality rates substantially decreased from 637/1000PY (95%CI: 551–736) in 2014 to 118/1000PY (95%CI: 110–126) in 2019 among Type 1 DM patients and from 253/1000PY (95%CI: 242–265) in 2014 to 136/1000PY (95%CI: 133–138) in 2019 among Type 2 DM patients (Fig. 3. Mortality rate per 100, 000 population/years (A) and per 1000 person-years (PY) with 95% CI (B).b).

### Comorbidities and diabetes-related complications stratified by duration of diabetes

**Fig. 4:**
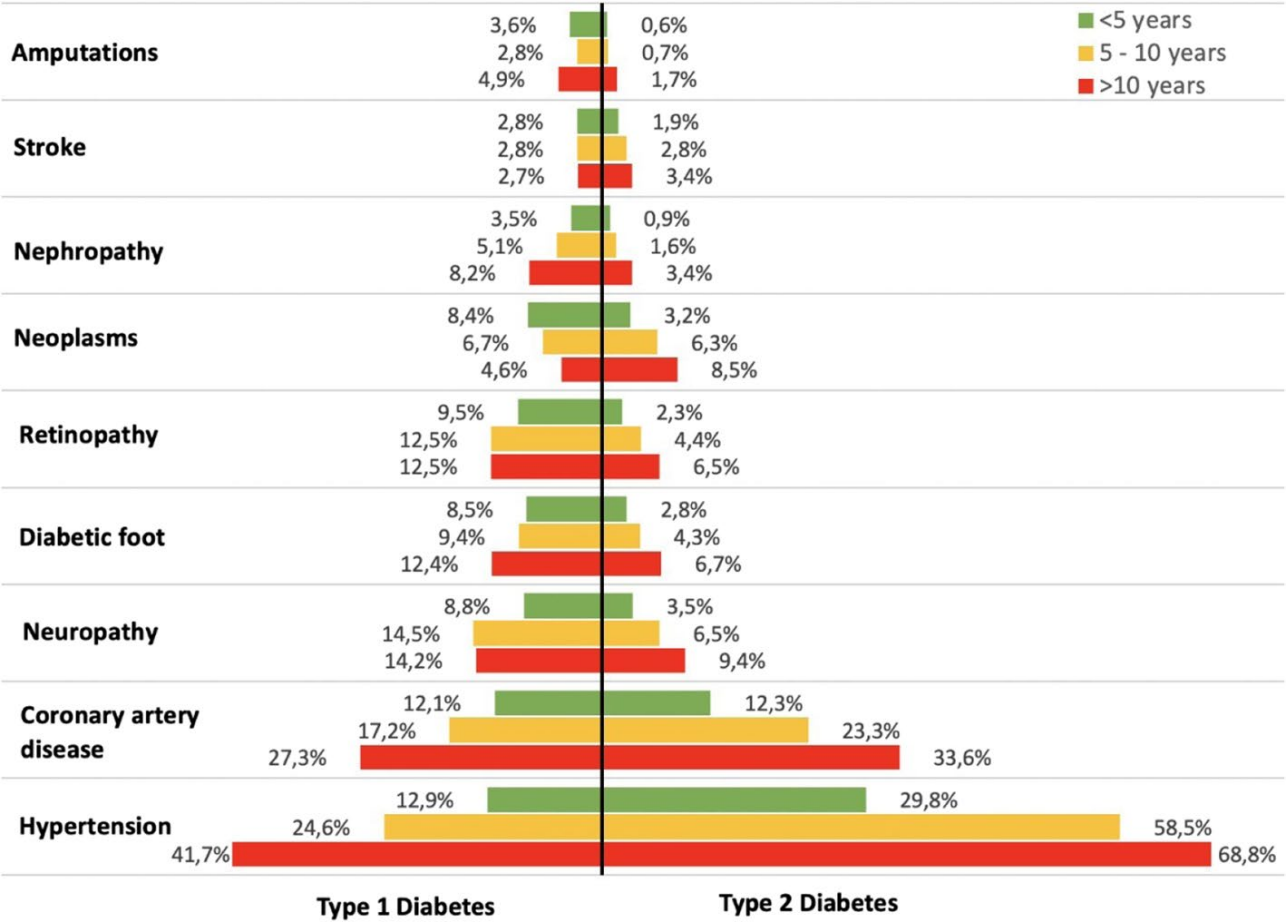
Prevalence of different comorbidities classified by type and duration of DM

In Fig. 4. Prevalence of different comorbidities classified by type and duration of DM., the prevalence of comorbidities and diabetes-related complications were stratified by the different duration of diabetes. The prevalence of amputations, stroke, nephropathy, and neoplasms showed fluctuations and did not exceed 10% among Type 1 DM patients with various disease duration, while among Type 2 DM patients with different disease duration consistent increase in prevalence was observed. Those patients with more than 10 years of Type 1 or Type 2 DM duration had higher prevalence of coronary artery disease (27.3 and 33.6%, respectively) and hypertension (41.7 and 68.8%, in turn) compared to patients with shorter disease duration.

### Association of age, sex and ethnicity with all-cause mortality

Median follow up was 4.7 (2.2–8.2) years and 4.5 (2.1–7.8) years for Type 1 and Type 2 DM, respectively. Male sex and age older than 18 were associated with a higher risk of all-cause death compared to female sex and younger age in patients with Type 1 and Type 2 DM (Supplementary Figs. 1-4). In adjusted models, males and advanced age categories among both Type 1 and Type 2 DM patients have higher risk of all-cause mortality (Table 2).

**Table 2:** Association of determinants with all-cause mortality in Type 1 and Type 2 DM patients

Variables	DM 1			DM 2		
	Crude HR (95%CI)	Adjusted HR (95%CI)	***p***-value	Crude HR (95%CI)	Adjusted HR (95%CI)	***p***-value
**Sex**			< 0.001			< 0.001
Female	1.0	1.0		1.0	1.0	
Male	0.96 (0.9–1.02)	1.36 (1.27–1.46)		1.36 (1.34–1.38)	1.64 (1.61–1.66)	
**Age**			< 0.001			< 0.001
< =18	1.0	1.0		1.0	1.0	
19–34	2.29 (1.98–2.65)	2.28 (1.97–2.63)		1.11 (0.83–1.47)	1.17 (0.88–1.56)	
35–50	4.69 (4.08–5.39)	4.79 (4.16–5.5)		2.16 (1.64–2.84)	2.32 (1.77–3.05)	
51–70	12.28 (10.74–14.03)	13.2 (11.57–15.1)		5.57 (4.24–7.31)	6.32 (4.81–8.31)	
> =71	37.39 (32.08–43.57)	42.4 (36.2–49.5)		19.21 (14.63–25.24)	22.7 (14.2–29.8)	
**Ethnicity**			< 0.001			< 0.001
Other	1.0	1.0		1.0	1.0	
Kazakh	0.87 (0.8–0.95)	1.18 (1.09–1.29)		0.9 (0.88–0.91)	1.02 (1.0–1.05)	
Russians	1.03 (0.94–1.12)	1.21 (1.11–1.32)		1.12 (1.1–1.14)	1.04 (1.02–1.06)	

*HR* hazard ratio, *DM 1* type 1 Diabetes mellitus, *DM 2* type 2 Diabetes mellitus

### Association of comorbidities and diabetes-related complications with all-cause mortality

In the multiple Cox regression (Table 3, Model 2) for Type 1 DM patients, the following comorbidities and diabetes-related complications as coronary artery disease (aHR 1.41; 95%CI 1.27–1.55), diabetic nephropathy (aHR 2.13; 95%CI 1.9–2.39), stroke (aHR 1.7; 95%CI 1.49–1.94), amputations (aHR 1.23; 95%CI 1.07–1.4) and neoplasms (aHR 1.28; 95%CI 1.13–1.45) were associated with a statistically significantly higher risk of all-cause mortality. In contrast, the following comorbidities and diabetes-related complications as diabetic neuropathy (aHR 0.79; 95%CI 0.68–0.92), hypertension (aHR 0.51; 95%CI 0.46–0.56), and diabetic retinopathy (aHR 0.68; 95%CI 0.57–0.8) were associated with a statistically significantly lower risk of all-cause mortality among Type 1 DM patients. Similarly, among Type 2 DM patients (Table 4, Model 2) having coronary artery disease (aHR 1.04; 95%CI 1.02–1.07), diabetic nephropathy (aHR 2.06; 95%CI 1.96–2.16), stroke (aHR 1.8; 95%CI 1.73–1.87), diabetic foot (aHR 1.23; 95%CI 1.18–1.28), amputations (aHR 1.64; 95%CI 1.54–1.75) and neoplasms (aHR 1.37; 95%CI 1.34–1.41) were associated with statistically significant increased risk of all-cause mortality, while diabetic neuropathy (aHR 0.81; 95%CI 0.77–0.85), hypertension (aHR 0.54; 95%CI 0.53–0.55), and diabetic retinopathy (aHR 0.72; 95%CI 0.68–0.77) were significantly associated with lower risk of all-cause mortality.

**Table 3:** Association of comorbidities with all-cause mortality in Type 1 DM patients

Variables	Crude HR and 95% CI	*p*-value	Model 1^a^ Adjusted HR and 95% CI	*p*-value	Model 2^b^ Adjusted HR and 95% CI	*p*-value
Diabetic neuropathy, yes	0.6 (0.53–0.67)	< 0.001	0.79 (0.7–0.89)	< 0.001	0.79 (0.68–0.92)	0.002
Coronary artery disease, yes	1.95 (1.82–2.09)	< 0.001	1.06 (0.99–1.14)	0.096	1.41 (1.27–1.55)	< 0.001
Hypertensionyes	1.39 (1.3–1.49)	< 0.001	0.68 (0.64–0.73)	< 0.001	0.51 (0.46–0.56)	< 0.001
Diabetic nephropathy, yes	1.92 (1.73–2.12)	< 0.001	1.71 (1.54–1.89)	< 0.001	2.13 (1.9–2.39)	< 0.001
Retinopathy, yes	0.53 (0.46–0.6)	< 0.001	0.75 (0.66–0.86)	< 0.001	0.68 (0.57–0.8)	< 0.001
Stroke, yes	3.0 (2.66–3.41)	< 0.001	1.67 (1.47–1.89)	< 0.001	1.7 (1.49–1.94)	< 0.001
Diabetic foot, yes	2.31 (2.13–2.5)	< 0.001	1.13 (1.04–1.23)	0.004	0.98 (0.88–1.09)	0.689
Amputations, yes	3.44 (3.12–3.81)	< 0.001	1.33 (1.2–1.48)	< 0.001	1.23 (1.07–1.4)	0.002
Neoplasms, yes	1.27 (1.12–1.44)	< 0.001	1.32 (1.17–1.5)	< 0.001	1.28 (1.13–1.45)	< 0.001

^a^Model 1 adjusted for sex, age categories and ethnicity

^b^Model 2 adjusted for sex, age categories, ethnicity, diabetic neuropathy, coronary artery disease, hypertension, diabetic nephropathy, retinopathy, stroke, diabetic foot, amputations and neoplasms

**Table 4:** Association of comorbidities with all-cause mortality in Type 2 DM patients

Variables	Crude HR and 95% CI	p-value	Model 1^a^ Adjusted HR and 95% CI	*p*-value	Model 2^b^ Adjusted HR and 95% CI	*p*-value
Diabetic neuropathy, yes	0.58 (0.56–0.6)	< 0.001	0.76 (0.74–0.79)	< 0.001	0.81 (0.77–0.85)	< 0.001
Coronary artery disease, yes	0.78 (0.77–0.8)	< 0.001	0.85 (0.84–0.87)	< 0.001	1.04 (1.02–1.07)	< 0.001
Hypertension, yes	0.56 (0.55–0.57)	< 0.001	0.58 (0.57–0.59)	< 0.001	0.54 (0.53–0.55)	< 0.001
Diabetic nephropathy, yes	1.26 (1.21–1.32)	< 0.001	1.63 (1.56–1.71)	< 0.001	2.06 (1.96–2.16)	< 0.001
Retinopathy, yes	0.53 (0.51–0.56)	< 0.001	0.72 (0.69–0.76)	< 0.001	0.72 (0.68–0.77)	< 0.001
Stroke, yes	1.5 (1.45–1.56)	< 0.001	1.56 (1.51–1.62)	< 0.001	1.8 (1.73–1.87)	< 0.001
Diabetic foot, yes	1.13 (1.1–1.18)	< 0.001	1.32 (1.27–1.36)	< 0.001	1.23 (1.18–1.28)	< 0.001
Amputations, yes	1.79 (1.69–1.88)	< 0.001	1.95 (1.85–2.05)	< 0.001	1.64 (1.54–1.75)	< 0.001
Neoplasms, yes	1.31 (1.28–1.35)	< 0.001	1.3 (1.27–1.34)	< 0.001	1.37 (1.34–1.41)	< 0.001

^a^Model 1 adjusted for sex, age categories and ethnicity

^b^Model 2 adjusted for sex, age categories, ethnicity, diabetic neuropathy, coronary artery disease, hypertension, diabetic nephropathy, retinopathy, stroke, diabetic foot, amputations and neoplasms

## Discussion

This is the first study from the Central Asian region to investigate the epidemiology of Type 1 and Type 2 DM patients using a large-scale administrative health data in Kazakhstan. In this study, we described incidence, prevalence and survival in Type 1 and Type 2 DM patients who were registered in UNEHS from 2014 to 2019 in Kazakhstan and investigated demographical factors and DM-related complications associated with all-cause mortality.

We reported that the incidence and prevalence of Type 1 DM in 2019 was 17 and 152 per 100,000, respectively, which was in line with reported numbers of International Diabetes Federation [12]. Recent meta-analysis by Mobasseri et al. also reported that the incidence of Type 1 diabetes in Asia, Africa, Europe, and America was 15 per 100,000, 8 per 100,000, 15 per 100,000 and 20 per 100, respectively. Also, the global prevalence of type 1 diabetes in the above regions was, 69 per 100,000, 35 per 100,000, and 122 per 100,000, respectively [13].

Our study reports the prevalence of Type 2 diabetes in 2019 of 2075 per 100,000 population. However, globally the prevalence of Type 2 diabetes estimated 6059 per 100,000, which was three times higher [14]. This finding shows a significant underestimation of the prevalence of Type 2 DM in Kazakhstan. In fact, a previous study based on multistage cluster random sampling showed that the prevalence of Type 2 Diabetes was 8% among 4753 participants from four distinct regions of Kazakhstan. And the same study reported a total of 54% of newly diagnosed Type 2 Diabetes patients [4]. Similar results were obtained from another study where authors reported the prevalence at 8.2% (95% CI 7.7%–8.6%) [5]. These findings suggest that a large proportion of patients are undiagnosed and unregistered. However, the trend of increasing prevalence of Type 2 DM over the years is in line with global trend [12, 15]. This trend can be partly explained by the rising life expectancy which may lead to increased prevalence of diabetes.

Mortality rate of Type 2 diabetes in our study was similar to WHO report 2016 which reports mortality rate 53.6 per 100,000 [16], while it was 55 per 100,000 in 2016 in Kazakhstan.

Diabetic neuropathy, nephropathy, retinopathy, diabetic foot and neoplasms were more common in Type 1 DM patients compared to Type 2 DM, while hypertension and coronary artery disease were more common in Type 2 DM patients compared to Type 1 DM.

Prevalence of coronary artery disease in our study was 18,7%, which was in line with global prevalence of 21% [12]. However, we reported lower prevalence of stroke (2.5%) compared to global prevalence (7.6%) in diabetic patients [12]. This might be due to undiagnosed and unregistered cases. Prevalence of diabetic retinopathy in Type 1 and Type 2 diabetes was 10,9% and 3,6%, respectively, which was within a wide range of global prevalence, 6.7 to 34.9% and 4.4 to 8.2%, respectively [12]. Prevalence of diabetic foot in our study was 5,4% with 1% suffering from amputation. These findings are in line with global prevalence of diabetic foot of 6,4% and amputations of 1% [12]. We reported a prevalence of diabetic nephropathy of 1.7% in Type 1 and Type 2 DM patients. However, the global prevalence of diabetic nephropathy is 30–50% [15, 17]. We may speculate that most cases registered in the database are advanced cases with end stage renal disease requiring dialysis. Nevertheless, the global prevalence of ESRD in patients with diabetes ranges from 19.0% in 2000 to 29.7% in 2015 worldwide [18]. Therefore, we might underestimate the prevalence of diabetic nephropathy.

We have found a significantly higher mortality rate in men compared to women with diabetes, which was also reported in the Kazakhstani general population (https://www.statista.com/statistics/974832/adult-mortality-rate-in-kazakhstan-by-gender/). Even though there is no data on men and women frequency of visits in Kazakhstan it might be that women comply to a larger extent than men with a doctor’s advice. A Swedish study showed that women with diabetes attend outpatient clinics more frequently than men with diabetes. That would give the women a greater opportunity to control high blood pressure and cholesterol levels [19]. The more frequent contact of women with health care in general may result in diabetes being diagnosed at an earlier and milder stage in women than in men, resulting in higher survival.

In contrast, Italian study reported the opposite findings of females having a higher risk of all-cause mortality than men (IRR 1.77; 95% CI 1.64–1.92) [18]. The authors explain this by the fact that females with diabetes have higher prevalent abdominal obesity, increasing the risk of hypertension and a worse lipid profile. The authors also reported that increased age is associated with increased risk of all-cause mortality in patients with diabetes [20]. This was in line with our findings.

Coronary artery disease, diabetic nephropathy, stroke, amputations and neoplasms were associated with a higher risk of all-cause death in patients with Type 1 and Type 2 DM. Diabetic neuropathy, hypertension, and diabetic retinopathy were associated with a lower risk of all-cause mortality in patients with Type 1 and Type 2 DM.

In the present study, non-hypertensive patients are at increased risk of all-cause mortality suggesting a paradoxical protective effect of elevated blood pressure in patients with Type 1 and Type 2 DM. This observation disagrees with the high risk of death conferred by elevated blood pressure in the general population. The apparent paradoxical protection afforded by elevated blood pressure against mortality observed in the present study can be partly explained by the phenomenon of reverse epidemiology of traditional risk factors reported in chronic conditions such as chronic kidney disease and dialysis, chronic heart failure, cancer and chronic infections like HIV/AIDS [21, 22]. This inverse relationship seems to indicate the existence of additional and more significant risk factors such as malnutrition and inflammation which might alter the relationship between traditional risk factors and outcomes in patients with diabetes [20, 21]. Another plausible explanation of the apparent paradox can be due to selection by indication. Registered hypertensive patients might be better protected and have a better track from healthcare professionals. Moreover, they may receive more intense medical care, including pharmacological therapy that may explain this association. However, data on medication was not available, so we could not explore this association.

The present study has several strengths. First, to our knowledge this is the first study from the Central Asian region to demonstrate the epidemiology of Type 1 and Type 2 DM patients based on a compiled nationwide digital healthcare data from Kazakhstan. Second, the data was linked to the Population Registry to determine the outcome data (died or alive).

Our study has a few limitations including the reliance on secondary data, which in turn is affected by the accuracy of measurement. Another main limitation is lack of data on treatments and clinical and laboratory data (blood pressure, HbA1c, cholesterol, etc.). Fair to mention also that in the current study the lack of cause-specific mortality data, possible errors with disease coding, and potential loss of follow-up for patients who might left the country could affect the results. Moreover, residual confounding factors (such as obesity, smoking status, lifestyle and socioeconomic factors, etc.) are likely present in the study results as the registry data was limited to few covariates to be adjusted for.

## Conclusion

The results showed an increase in the prevalence and mortality but not the incidence in patients with Type 1 and Type 2 DM. We described that male sex, older age and Kazakh ethnicity were associated with a higher risk of all-cause death compared to females, younger age and other nationalities than Kazakh in patients with Type 1 and Type 2 DM. We also described that coronary artery disease, diabetic nephropathy, stroke, amputations and neoplasms were associated with a higher risk of all-cause death in patients with Type 1 and Type 2 DM. Diabetic neuropathy, hypertension, and diabetic retinopathy were associated with a lower risk of all-cause mortality in patients with Type 1 and Type 2 DM.

## Supplementary Information

**Additional file 1.**

## Data Availability

The data that support the findings of this study are available from Republican Center for Electronic Health of the Ministry of Health of the Republic of Kazakhstan, but restrictions apply to the availability of these data, which were used under the contract-agreement (#173–2020, from March 11th, 2020) for the current study, and so are not publicly available. Data are however available from the authors upon reasonable request and with permission of Ministry of Health of the Republic of Kazakhstan. For those who want to request the data from this study, contact the Principal Investigator of the research grant Dr. Abduzhappar Gaipov (Email: abduzhappar.gaipov@nu.edu.kz, phone + 77172706297).
