# Supplementary material for: Epidemiology of type 1 and type 2 diabetes mellitus in Kazakhstan: data from unified National Electronic Health System 2014–2019
Source: BMC Endocr Disord. 2022 Nov 11;22:275. doi: 10.1186/s12902-022-01200-6 (PMC9650815; doi:10.1186/s12902-022-01200-6)

Supplementary materials

Supplementary Table 1 ICD-9 and ICD-10 codes used for comorbidities in Type 1 and Type 2 DM patients

**Comorbidities**	**ICD-9p**	**ICD 10**
Coronary artery disease	00.66, 36.00-36.99	I20-I25
Hypertension	-	I10-I16
Stroke	00.61, 00.62, 00.64, 00.65	I60-I66
Diabetic retinopathy	14.0-14.9	E10.3, E11.3, E12.3, E13.3, E14.3, H25-H26, H28, H30-H36, H43.1, H54
Diabetic nephropathy	55.6	E10.2, E11.2, E12.2, E13.2, E14.2, N17.0–N19.9, Z99.2
Diabetic neuropathy	-	E10.4, E11.4, E12.4, E13.4, E14.4, G63.2, G62.9
Diabetic foot	-	E10.5, E11.5, E12.5, E13.5, E14.5, L97, L98.4, R02, M86
Amputations	84.1	-
Neoplasms	-	C00-D49

Supplementary Figure 1. Kaplan-Meier survival analysis of all-cause mortality in Type 1 DM patients classified by sex, adjusted for age and ethnicity

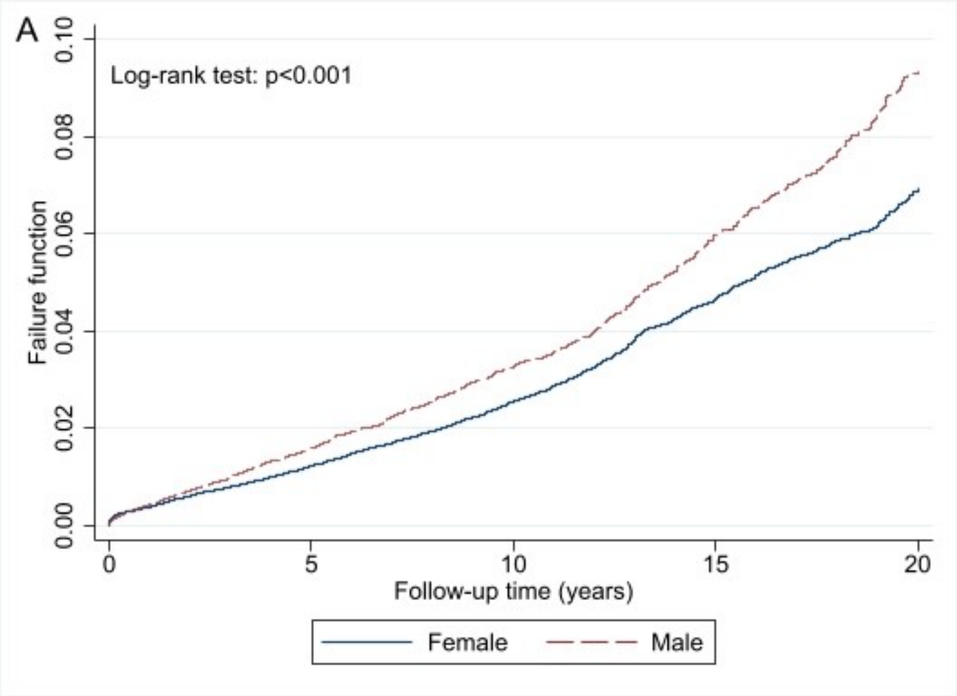

Supplementary Figure 2. Kaplan-Meier survival analysis of all-cause mortality in Type 1 DM patients classified by age groups, adjusted for sex and ethnicity.

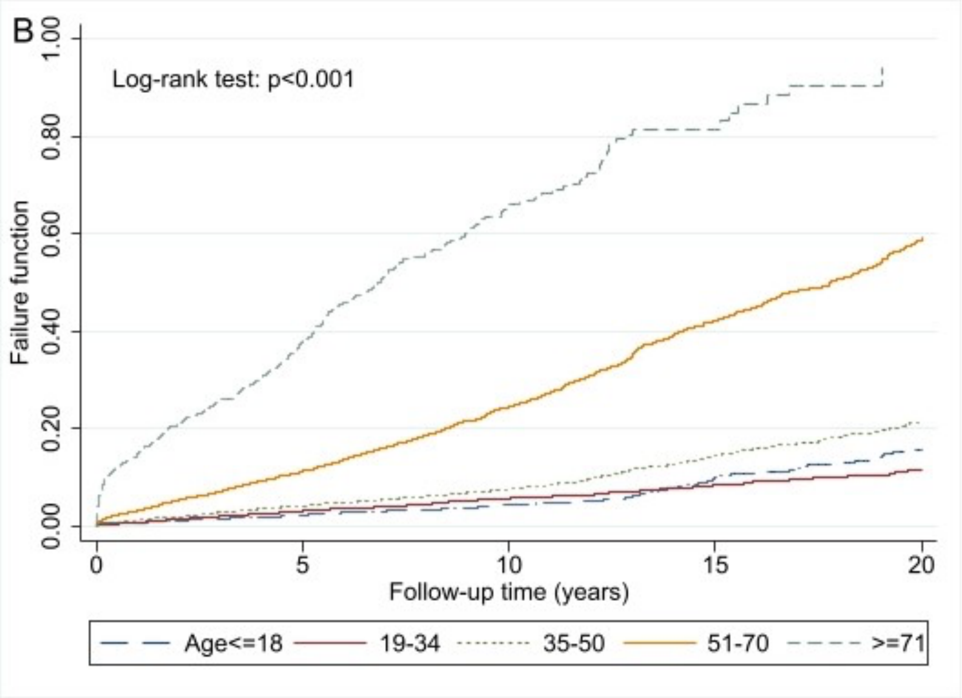

Supplementary Figure 3. Kaplan-Meier survival analysis of all-cause mortality in Type 2 DM patients classified by sex, adjusted for adjusted for age and ethnicity.

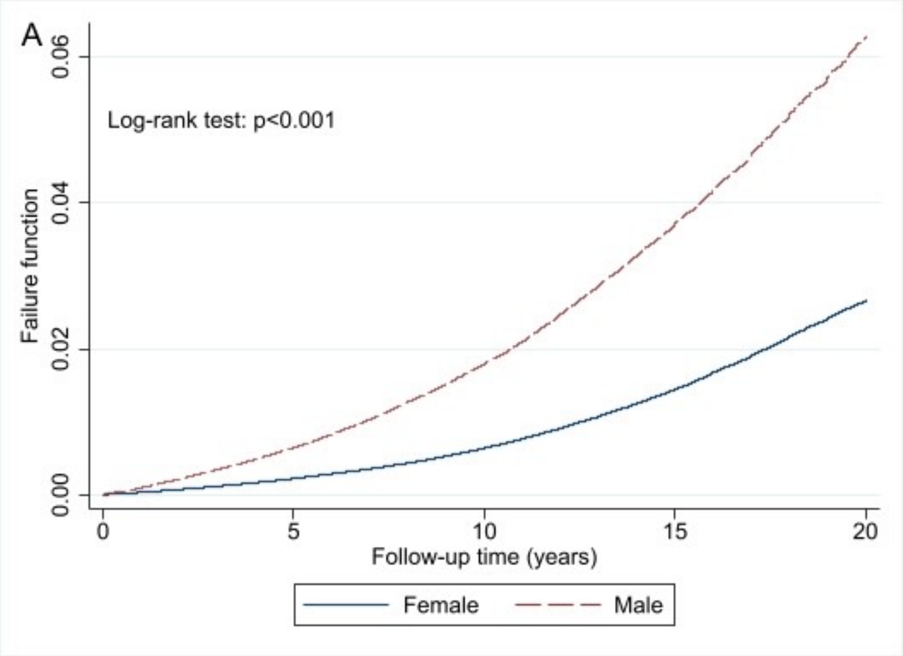

Supplementary Figure 4. Kaplan-Meier survival analysis of all-cause mortality in Type 2 DM patients classified by age groups, adjusted for sex and ethnicity.

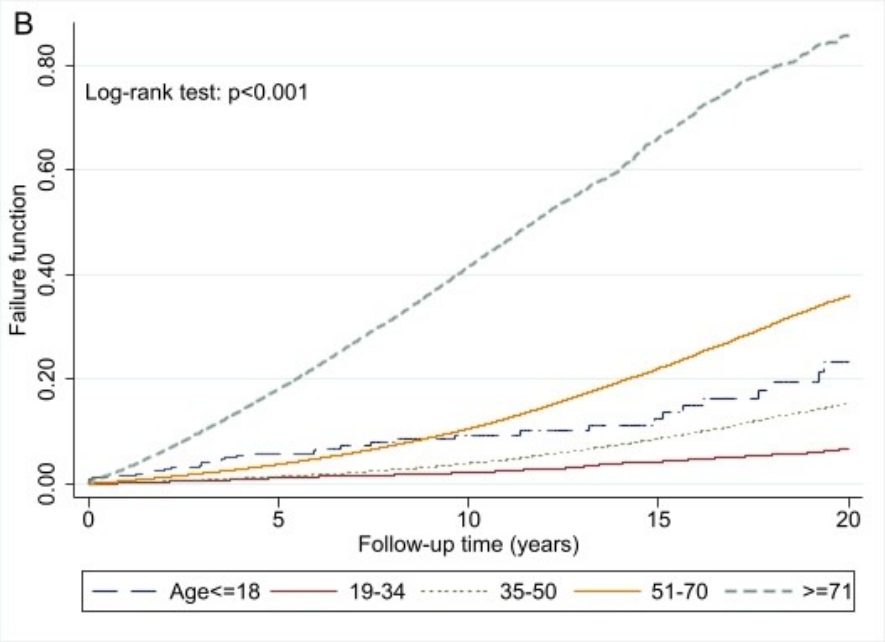

Supplement: Supplementary file 1 — Additional file 1. [file 12902_2022_1200_MOESM1_ESM.pdf]
